# Interaction between dietary total antioxidant capacity and BDNF Val66Met polymorphism on lipid profiles and atherogenic indices among diabetic patients

**DOI:** 10.1038/s41598-021-98663-9

**Published:** 2021-09-27

**Authors:** Faezeh Abaj, Masoumeh Rafiee, Fariba Koohdani

**Affiliations:** 1 Department of Community Nutrition, School of Nutritional Sciences and Dietetics, Tehran University of Medical Sciences, Tehran, Iran; 2 Department of Clinical Nutrition, School of Nutrition and Food Science, Isfahan University of Medical Sciences (IUMS), Isfahan, Iran; 3 Department of Cellular, Molecular Nutrition, School of Nutritional Sciences and Dietetics, Tehran University of Medical Sciences (TUMS), PO Box: 141556117, Tehran, Iran

**Keywords:** Genetic interaction, Medical genetics, Cardiovascular diseases, Nutrition disorders, Genetics, Molecular medicine

## Abstract

Brain-derived neurotrophic factor (BDNF) belongs to the “neurotrophin” family of growth factors, and it has recently been associated to cardiovascular disease (CVD). We anticipated that BDNF Val66Met polymorphisms may alter CVD risk markers such as serum lipid profile differences, and interaction with total antioxidant capacity of diet (DTAC) could alter these clinical parameters. This cross-sectional study consisted of 667 diabetic patients (39.7% male and 60.3% female). DTAC was calculated by international databases. Biochemical markers including total cholesterol (TC), low-density lipoprotein (LDL), high-density lipoprotein (HDL), triglyceride (TG), superoxide dismutase (SOD), C-reactive protein (CRP), total antioxidant capacity (TAC), pentraxin-3 (PTX3), isoprostaneF2α (PGF2α). interleukin 18 (IL18), leptin and ghrelin were measured by standard protocol. Atherogenic indices (AIP, AC, CR-I, CR-II) were calculated. Genotyping of the BDNF Val66Met polymorphisms was conducted by the real-time PCR–RFLP method. The gene-diet interactions were evaluated using a generalized linear mode (GLMs). Carriers of the Val/Met genotype who were in the higher median intake of FRAP had lower HDL (P:0.04) and higher TG (P:0.005), AIP (P:0.02) and AC (P:0.02) index compared to Val/Val genotypes with lower median intake. Moreover, diabetic patients with Val/Met genotype who consumed higher ORAC intake had increased odds for anthropometric indices (BMI (P:0.01) and WC (P:0.03)), lipid profiles (TG) (P:0.01), and atherogenic index (AIP) (P:0.02), also decreased odds for HDL (P:0.03) concentration compared to reference group whit lower ORAC intake. Individuals with Val/Met genotype who consumed higher TRAP intake had increased odds for WC (P:0.04), TC (P:0.001), TG (P < 0.001), AIP (P < 0.001) and AC (P < 0.001). Finally, Val/Met patients with a higher median intake of TEAC had higher TG (P:0.02), AIP (P:0.009) and AC (P:0.03) compared to the reference group whit lower TEAC intake. Our study showed that Val/Met genotype had also the highest lipid profile and atherogenic indices even in the highest adherence to DTAC. While it seems that the presence of the Val/Val wild-type and BDNF Met/Met homozygotes in diabetic patients with a high DTAC is a protective factor.

## Introduction

High lipid serum concentrations are involved in the development of CVD and metabolic disorders. As a result, lipid level detection and treatment are critical in preventing CVD and its implications in diabetes patients^1,2^. Significant evidence suggests that high TC and TG levels, as well as an inverse association with HDL-C, are directly or indirectly connected to the risk of CVD^3,4^. The TC/HDL ratio is a more specific and sensitive indicator of cardiovascular risk than TC, with a value more than 5.5 indicating significant atherogenic risk^5^. The atherogenic index of plasma, in addition to dyslipidemia, is one of the most powerful markers in predicting the risk of CVD. The atherogenic index of plasma (AIP) is a new index^6–8^, that has been used to quantify blood lipid levels and is often utilized as an optimum indication of dyslipidemia and related problems such as CVD^9–11^.

Environmental factors, such as dietary consumption, genetic variations, and their interactions, have an impact on blood lipid levels^12–15^. Dietary changes could impact the oxidation profile of patients with various clinical diseases including diabetes, dyslipidemia, and CVDs, which are affected by foods and diets including polyphenols, flavonoids, PUFAs, and the Mediterranean diet^16,17^. The influence of antioxidants alone in decreasing oxidative stress has been identified; however, combining antioxidants is shown to have a synergistic activity; the perfect way to demonstrate this is to estimate the cumulative function and complex effects of all antioxidants in body fluids; total antioxidant capacity (TAC) is characterized as the moles of a given free radical removed by a test solution^16,18,19^. According to studies, DTAC is inversely connected to cancer fatalities, cardiovascular disease deaths, and all other causes of death^20^. Given the impact of DTAC on the aforementioned parameters, it's not unreasonable to believe that DTAC is related to atherogenic and anthropometric indices.

Moreover, BDNF is a candidate gene among the genetic variables linked to serum lipid levels that have been reported. The human BDNF gene is found on chromosome 11, in the region p13–14, and is mainly expressed in brain tissue^21,22^. The valine (Val) to a methionine (Met) substitution at codon 66 of BDNF gene, which can disrupt construction of BDNF into secretory vesicles and lead to low secretion of BDNF^23^. Several studies have showed the possible association between Met allele and insulin resistance and obesity in patients with schizophrenia^24^. In study of Leipzig Childhood cohort on German obese children showed that the Met allele associated with lower body mass index (BMI), postprandial glucose and HbA1c levels, however had not significant association with serum insulin and lipid profile^25^. The effects of BDNF single-nucleotide polymorphisms (SNPs) on the risk of cardiovascular disease, type 2 diabetes, and metabolic syndrome have been investigated extensively^26–28^. Gene variations have been found to interact with nutritional intakes, eating preferences, and body composition in several investigations^29,30^. In some studies, met-allele carriers were directly correlated to the high intake of dietary total calorie, carbohydrate, and protein.

In Puerto Rican men, the BDNF rs6265 GG genotype has been related to increased, but in women, it has been related to a lower BMI. However the reasons for the gender disparities are unknown, they appeared to be related to variations in PUFA intake, and there was a significant interplay between the BDNF alleles and u-3 and u-6 PUFA consumption^30^. As a result, the interactions between the rs6265 BDNF variation and ethnic and nutritional factors appear to be essential, but they are still unknown. Although there are some nutrigenetic studies related to DTAC, to the best of our knowledge, no studies have investigated the interactions of BDNF variants with dietary TAC on atherogenic indices changes. Hence, this study aimed to find out the possible relationship between this dietary index and atherogenic indices and compare it among BDNF Val/Met polymorphism groups.

## Result

### Study population characteristics

In the current study, 667 patients with T2DM were evaluated, sex distribution of the population was 39.7% and 60.3% male and female, respectively, 17.5% were smoker and 81.8% had a family history of diabetes. The means and standard deviation (SD) of age, BMI, and WC of individuals were (54.03 ± 6.51 years, 29.42 ± 4.64 kg/m^2^, and 92.67 ± 10.72 cm), respectively. According to our findings, genotype distribution of BDNF Val66Met (rs6265) in type 2 diabetes population was 54.9%, 35.2% and 9.9% for Val/Val, Val/Met and Met/Met. Genotype frequencies were in Hardy Weinberg equilibrium (P > 0.05). Also, the median intake of DTAC was (FRAP (15.83) TRAP (8.19) TEAC (7.46), and ORAC (27,372.14)).

### Association between population characteristics, biochemical parameters between DTAC and BDNF Val66Met polymorphism

We found that there was no significant association between lipid profiles and atherogenic indices among BDNF Val66Met genotypes (p > 0.05**) (**Table 1).

**Table 1:** The association between BDNF Val/Met polymorphism with lipid profiles and atherogenic indices in T2DM patients.

	BDNF Val/Met polymorphism			P-value
	Val/Val	Val/Met	Met/Met	
Age (year)	54.20 ± 6.28	53.91 ± 6.97	53.56 ± 6.19	0.71
Sex (male) N%	155(58.5%)	81(30.6%)	29(10.9%)	0.11
Cigarette smoking (yes) N%	76 (58.5%)	37 (28.5%)	17 (13.1%)	0.12
Alcohol consumption (no) N%	355 (55%)	229 (35.4%)	62 (9.6%)	0.34
Familial history of diabetes (yes) N%	296 (54.9%)	186 (34.5%)	57 (10.6%)	0.42
Glucose-lowering medication	297 (53.9%) 69 (59.5%)	197 (35.8%) 38 (32.8%)	57 (10.3%) 9 (7.8%)	0.48
Metformin and glybenclamid N%				
Other medications N%				
Supplementation use (yes)	364 (55%)	232 (35%)	66 (10%)	0.45
Total energy intake, kcal/day	2606.74 ± 1030.58	2627.05 ± 918.05	2537.43 ± 865.26	0.80
BMI (kg/m^2^)	29.19 ± 4.61	29.78 ± 4.86	29.38 ± 3.86	0.30
WC (cm)	92.67 ± 10.90	92.70 ± 10.61	92.62 ± 10.21	0.99
HDL (mg/dl)	54.95 ± 13.40	54.66 ± 12.11	55.83 ± 11.32	0.80
LDL (mg/dl)	109.43 ± 34.33	112.48 ± 37.13	116.50 ± 38.50	0.27
LDL/HDL	3.03 ± 13.16	2.11 ± 0.67	2.12 ± 0.65	0.48
TC (mg/dl)	193.06 ± 65.66	199.80 ± 75.98	199.72 ± 66.06	0.46
TG (mg/dl)	183.77 ± 106.1	179.10 ± 103.24	192.46 ± 138.80	0.67
Leptin (ng/ml)	24.28 ± 13.55	27.67 ± 16.54	23.01 ± 14.18	0.16
Ghrelin (ng/ml)	2.15 ± 1.18	2.37 ± 1.30	2.33 ± 1.21	0.41
AIP	0.47 ± 0.23	0.46 ± 0.24	0.45 ± 0.27	0.82
AC	2.68 ± 1.51	2.81 ± 1.62	2.64 ± 1.17	0.54
CRI.II	2.06 ± 0.69	2.11 ± 0.67	2.12 ± 0.65	0.63
CRI-I	3.80 ± 1.67	3.83 ± 1.46	3.51 ± 1.05	0.32
FRAP (mmol Fe^2+^/100 g)	18.17 ± 13.41	17.35 ± 7.72	16.95 ± 6.62	0.56
TRAP (mmol TE/kg)	9.68 ± 5.56	9.75 ± 5.62	8.65 ± 3.70	0.32
TEAC (mmol TE/kg)	8.63 ± 4.30	8.63 ± 4.14	7.98 ± 3.09	0.49
ORAC (µmol TE/100 g)	30,553.29 ± 16,659.94	29,889.21 ± 12,158.26	29,602.80 ± 10,295.74	0.81

Data are presented as mean ± standard deviation (SD).

*BMI* body mass index, *HDL-c* high density lipoprotein cholesterol, *LDL-c* low density lipoprotein cholesterol, *TG* triglyceride, *CRP* C-reactive protein, *PTX3* pentraxin-3, *IL18* interleukin 18, *TAC* total antioxidant capacity, *SOD* superoxide dismutase, *PGF2α* prostaglandinF2α, *FRAP* ferric reducing ability of plasma, *TRAP* total reactive antioxidant potential, *TEAC* trolox equivalent antioxidant capacity, *ORAC* oxygen radical absorbance capacity, *AIP* log (TG/HDL), *AC* (TC-HDL)/HDL, *CRI.II* (LDL/HDL), *CRI-I* (TC/HDL).

Lipid profiles and atherogenic indices among DTAC groups (ferric reducing-antioxidant power (FRAP), total radical-trapping antioxidant parameter (TRAP), total reactive antioxidant potential (TEAC), and oxygen radical absorbance capacity (ORAC)), is presented in Tables 2 and 3. An individual with higher adherence to FRAP (P = 0.02), TEAC (P = 0.04), and ORAC (P = 0.01) had lower TAC concentrations. Moreover, patients with a higher intake of TRAP (P = 0.03) were more likely to have higher IL-18 concentrations.

**Table 2:** The association between lipid profiles and atherogenic indices with DTAC (FRAP, TRAP) in T2DM patients.

	FRAP Mean ± SD		P	TRAP Mean ± SD		P
	Low	High		Low	High	
	327	321		335	325	
Age (year)	53.83 ± 6.53	54.13 ± 6.36	0.55	54.06 ± 6.68	53.86 ± 6.28	0.68
Sex (male) N%	127 (49.2%)	131 (50.8%)	0.33	129 (49%)	134 (51%)	0.28
BMI (kg/m^2^)	29.42 ± 4.96	29.42 ± 4.36	0.98	29.43 ± 4.88	29.39 ± 4.40	0.90
WC (cm)	92.63 ± 10.64	92.89 ± 10.92	0.75	92.99 ± 11.20	92.38 ± 10.25	0.46
Occupation unemployed	168 (51.9%)	156 (48.1%)	0.26	158 (48.2%)	170 (51.8%)	0.27
Education university educated	67 (54%)	57 (46%)	0.21	73 (58.4%)	52 (41.6%)	**0.03**
Cigarette smoking (yes) N%	50 (40%)	75 (60%)	**0.006**	54 (42.2%)	74(57.8%)	**0.02**
Alcohol consumption (NO) N%	317 (50.6%)	310 (49.4%)	0.48	323 (50.5%)	316 (49.5%)	0.35
Familial history of diabetes (yes) N%	227 (50.3%)	224 (49.7%)	0.49	235 (51.2%)	224 (48.8%)	0.39
Glucose-lowering medication	279 (52.1%) 48 (42.58%)	256 (47.9%) 65 (57.5%)	**0.03**	283 (51.9%) 52 (45.2%)	262 (48.1%) 63 (54.8%)	0.11
Metformin and glybenclamid N%						
Other medications N%						
Supplementation use (yes)	326 (50.7%)	317 (49.3%)	0.18	334 (51%)	321 (49%)	0.17
Total energy intake, kcal/day	2594.64 ± 961.68	2639. ± 998.72	0.53	2569.34 ± 950.53	2654.10 ± 999.75	0.23
Carbohydrate, g/day	341.16 ± 141.08	352.74 ± 148.18	0.27	337.21 ± 131.41	355.31 ± 155.02	0.08
Protein, g/day	90.55 ± 35.87	91.73 ± 37	0.65	90.30 ± 35.60	91.72 ± 37.12	0.58
Total fat, g/day	104.83 ± 51.36	103.60 ± 47.07	0.73	104.06 ± 50.50	103.95 ± 47.60	0.97
Saturated fatty acids, g/day	26.70 ± 10.40	27.15 ± 12.19	0.58	26.49 ± 10.55	27.25 ± 11.98	0.35
Monounsaturated fatty acids, g/day	36.14 ± 19.79	35 ± 17.35	0.39	35.91 ± 19.28	35.29 ± 17.76	0.75
Polyunsaturated fatty acids, g/day	25.83 ± 16.29	25.43 ± 14.16	0.71	25.45 ± 15.55	25.73 ± 14.85	0.79
Cholesterol, (mg/day)	222.69 ± 143.12	221.49 ± 49	0.93	226.41 ± 160.26	221.60 ± 22.07	0.73
Dietary fiber, g/day	41.64 ± 21.86	44.28 ± 24.30	0.11	41.12 ± 12.34	44.58 ± 24.43	**0.03**
HDL (mg/dl)	54.48 ± 12.48	55.28 ± 13.05	0.43	54.93 ± 12.25	54.86 ± 13.24	0.94
LDL (mg/dl)	111.33 ± 35.51	110.50 ± 35.81	0.76	111.24 ± 35.60	110.69 ± 35.65	0.84
Cholesterol (mg/dl)	194.92 ± 69.25	195.40 ± 70.33	0.93	196.81 ± 68.23	194.15 ± 70.93	0.62
TG (mg/dl)	181.05 ± 110.92	182.38 ± 105.97	0.87	184.39 ± 116.49	180.72 ± 100.36	0.66
Leptin (ng/ml)	25.28 ± 13.58	25.45 ± 15.88	0.92	25.10 ± 13.70	25.29 ± 15.77	0.91
Fat%	36 ± 8.39	34.79 ± 7.63	0.05	35.85 ± 7.88	34.93 ± 8.19	0.14
Ghrelin (ng/ml)	2.19 ± 1.24	2.27 ± 1.25	0.61	2.22 ± 1.30	2.26 ± 1.65	0.77
CRP (mg/l)	2.06 ± 1.39	2.20 ± 1.57	0.56	2.12 ± 1.47	2.19 ± 1.50	0.76
Pentrexin3 (ng/ml)	2.63 ± 0.52	2.56 ± 0.39	0.36	2.62 ± 0.45	2.59 ± 0.46	0.70
Interlukin18 (pg/ml)	247.61 ± 32.24	248.68 ± 27.86	0.82	241.96 ± 32.05	252.24 ± 27.71	**0.03**
TAC (g/dl)	2.62 ± 0.58	2.40 ± 0.56	**0.02**	2.60 ± 0.59	2.44 ± 0.55	0.08
SOD (U/ml)	0.14 ± 0.04	0.15 ± 0.10	0.52	0.15 ± 0.04	0.16 ± 0. 08	0.65
PGF2alpha (pg/ml)	72.48 ± 6.46	72.52 ± 5.61	0.96	72.50 ± 6.39	72.38 ± 5.83	0.90
AIP	0.46 ± 0.25	0.47 ± 0.23	0.72	0.46 ± 0.25	0.47 ± 0.23	0.77
AC	2.73 ± 1.56	2.69 ± 1.49	0.74	2.72 ± 1.53	2.70 ± 1.51	0.86
CRI.II	2.10 ± 0.68	2.05 ± 0.66	0.36	2.08 ± 0.67	2.08 ± 0.68	0.93
CRI-I	3.83 ± 1.54	3.79 ± 1.57	0.74	3.76 ± 1.52	3.82 ± 1.59	0.61

Data are presented as mean ± standard deviation (SD). Bold values denote statistical signifcance at the P < 0.05 level.

*BMI* body mass index, *HDL-c* high density lipoprotein cholesterol, *LDL-c* low density lipoprotein cholesterol, *TG* triglyceride, *CRP* C-reactive protein, *PTX3* pentraxin-3, *IL18* interleukin 18, *TAC* total antioxidant capacity, *SOD* superoxide dismutase, *PGF2α* prostaglandinF2α, *AIP* log (TG/HDL), *AC* (TC-HDL)/HDL, *CRI.II* (LDL/HDL), *CRI-I* (TC/HDL).

**Table 3:** The association between lipid profiles and atherogenic indices with DTAC (TEAC, ORAC) in T2DM patients.

	TEAC Mean ± SD		P	ORAC Mean ± SD		P
	Low	High		Low	High	
Age (year)	53.95 ± 6.61	54.01 ± 6.34	0.89	54.02 ± 6.75	53.90 ± 6.22	0.80
Sex (male) N%	131 (49.8%)	132 (50.2%)	0.48	123 (46.9%)	139 (53.1%)	0.16
BMI (kg/m^2^)	29.29 ± 4.88	29.51 ± 4.41	0.54	29.36 ± 4.79	29.49 ± 4.54	0.72
WC (cm)	92.72 ± 11.19	92.68 ± 10.29	0.95	92.54 ± 10.68	92.93 ± 10.83	0.64
Occupation unemployed N%	166 (50.5%)	163 (49.5%)	0.45	158 (48.2%)	170 (51.8%)	0.27
Education university educated N%	64 (51.2%)	61 (48.8%)	0.42	70 (56.5%)	54 (43.5%)	0.05
Cigarette smoking (yes) N%	55 (40.3%)	73 (57%)	**0.04**	54 (41.9%)	75 (58.1%)	**0.03**
Alcohol consumption (NO) N%	321 (50.3%)	317 (49.7%)	0.32	316 (49.7%)	320 (50.3%)	0.34
Familial history of diabetes (yes) N%	267 (50%)	267 (50%)	0.44	224 (49.1%)	232 (50.9%)	0.42
Glucose-lowering medication	248 (52.2%) 46 (40%)	260 (47.8%) 69 (60%)	**0.01**	281 (51.8%) 44 (38.3%)	261 (48.2%) 71 (61.7%)	**0.005**
Metformin and glybenclamid N%						
Other medications N%						
Supplementation use (yes) N%	329 (50.3%)	325 (49.95)	0.18	325 (49.8%)	327(50.2%)	0.03
Total energy intake, kcal/day	2555.76 ± 946.78	2670.21 ± 1002.04	0.10	2572.94 ± 926.81	2654.90 ± 1023.31	0.24
Carbohydrate, g/day	336.14 ± 131.80	356.64 ± 154.67	**0.04**	330.73 ± 130.61	353.17 ± 154.60	**0.03**
Protein, g/day	90.16 ± 35.48	91.96 ± 37.23	0.49	90.05 ± 34.30	92.10 ± 38.35	0.43
Total fat, g/day	103.27 ± 50.12	104.86 ± 47.99	0.65	103.13 ± 49.99	105.15 ± 48.18	0.56
Saturated fatty acids, g/day	26.53 ± 10.82	27.22 ± 11.75	0.39	26.60 ± 10.35	27.16 ± 12.17	0.49
Monounsaturated fatty acids, g/day	35.52 ± 19.17	35.53 ± 19.17	0.99	35.38 ± 19.04	35.73 ± 18.06	0.79
Polyunsaturated fatty acids, g/day	25.31 ± 15.23	25.90 ± 15.18	0.59	25.11 ± 15.68	26.15 ± 14.73	0.34
Cholesterol, (mg/day)	222.44 ± 144.44	225.65 ± 232.93	0.81	218.93 ± 122.38	229.43 ± 245.65	0.45
Dietary fiber, g/day	41.18 ± 21.44	44.57 ± 24.37	**0.04**	42.06 ± 12.75	43.79 ± 24.24	0.29
HDL (mg/dl)	54.79 ± 12.33	55.02 ± 13.16	0.81	54.68 ± 12.50	55.07 ± 12.97	0.69
LDL (mg/dl)	111.91 ± 36.23	110.04 ± 35.03	0.50	110.52 ± 35.30	111.49 ± 36.08	0.72
Cholesterol (mg/dl)	196.97 ± 69.42	193.92 ± 69.79	0.57	194.26 ± 71.39	196.32 ± 67.68	0.70
TG (mg/dl)	185.42 ± 113.34	178.96 ± 103.33	0.45	184.29 ± 111.44	179.20 ± 105.06	0.55
Leptin (ng/ml)	25.19 ± 13.75	25.20 ± 15.57	0.99	24.82 ± 13.74	25.56 ± 15.62	0.68
Fat%	35.65 ± 7.81	35.15 ± 8.28	0.42	35.54 ± 8.08	35.30 ± 8.05	0.71
Ghrelin (ng/ml)	2.11 ± 1.12	2.35 ± 1.31	0.13	2.18 ± 1.19	2.29 ± 1.27	0.51
CRP (mg/l)	2.08 ± 1.47	2.23 ± 1.50	0.55	2.13 ± 1.36	2.15 ± 1.58	0.92
Pentrexin3 (ng/ml)	2.65 ± 0.50	2.56 ± 0.42	0.21	2.63 ± 0.52	2.57 ± 0.39	0.44
Interlukin18 (pg/ml)	247.35 ± 27.85	247.97 ± 32.16	0.89	245.56 ± 32.45	250.29 ± 27.33	0.33
TAC (g/dl)	2.60 ± .58	2.42 ± .55	**0.04**	2.62 ± 0.58	2.39 ± 0.55	**0.01**
SOD (U/ml)	0.15 ± 0.04	0.15 ± 0.09	0.85	0.14 ± 0.04	0.15 ± 0.10	0.33
PGF2alpha (pg/ml)	72.29 ± 6.22	72.56 ± 5.95	0.78	72.33 ± 6.49	72.50 ± 5.67	0.86
AIP	0.47 ± 0.24	0.46 ± 0.24	0.64	0.47 ± 0.25	0.46 ± 0.23	0.74
AC	2.73 ± 1.54	2.68 ± 1.49	0.66	2.69 ± 1.57	2.73 ± 1.47	0.77
CRI.II	2.09 ± .67	2.06 ± 0.67	0.56	2.07 ± 0.66	2.09 ± 0.68	0.80
CRI-I	3.81 ± 1.75	3.78 ± 1.32	0.80	3.80 ± 1.54	3.80 ± 1.56	0.98

Data are presented as mean ± standard deviation (SD). Bold values denote statistical signifcance at the P < 0.05 level.

*BMI* body mass index, *HDL-c* high density lipoprotein cholesterol, *LDL-c* low density lipoprotein cholesterol, *TG* triglyceride, *CRP* C-reactive protein, *PTX3* pentraxin-3, *IL18* interleukin 18, *TAC* total antioxidant capacity, *SOD* superoxide dismutase, *PGF2α* prostaglandinF2α, *AIP* log (TG/HDL), *AC* (TC-HDL)/HDL, *CRI.II* (LDL/HDL), *CRI-I* (TC/HDL).

### Interaction between the BDNF Val66Met polymorphism and DTAC

Tables 4, 5, 6, 7 show the interactions between BDNF Val66Met polymorphism and DTAC (FRAP, TRAP, TEAC, and ORAC) on anthropometric indices (BMI and WC), lipid profiles (HDL, LDL, TC, and TG), and atherogenic indices (AIP, AC, CRI.II and CRI).

**Table 4:** The interaction of BDNF Val/Met polymorphism and FRAP on anthropometric indices, lipid profile and atherogenic indices.

Variable		Allele	High adherence of FRAP		
			β	95% CI	P
BMI	Crud	Met/Met	0.55	− 1.94 to 3.04	0.66
		Val/Met	0.66	− 0.88 to 2.21	0.40
		Val/Val	Reference		
	Adjusted	Met/Met	0.64	− 1.85 to3.12	0.61
		Val/Met	0.72	− 0.81 to 2.26	0.35
		Val/Val	Reference		
WC	Crud	Met/Met	− 0.15	− 5.92 to 5.60	0.86
		Val/Met	0.48	− 3.11 to 4.07	0.79
		Val/Val	Reference		
	Adjusted	Met/Met	− 0.48	− 6.09 to 5.11	0.86
		Val/Met	0.23	− 3.25 to 3.73	0.89
		Val/Val	Reference		
HDL	Crud	Met/Met	− 7.25	− 14.04 to − 0.47	**0.03**
		Val/Met	− 4.39	− 8.63 to − 0.16	**0.04**
		Val/Val	Reference		
	Adjusted	Met/Met	− 6.10	− 12.69 to 0.49	0.07
		Val/Met	− 4.18	− 8.29 to − 0.68	**0.04**
		Val/Val	Reference		
TG	Crud	Met/Met	25.52	− 32.43 to 83.48	0.86
		Val/Met	49.98	13.86 to 86.11	**0.007**
		Val/Val	Reference		
	Adjusted	Met/Met	24.16	− 34.37 to 82.71	0.41
		Val/Met	51.84	15.39 to 88.30	**0.005**
		Val/Val	Reference		
LDL	Crud	Met/Met	− 1.66	− 20.67 to 17.33	0.43
		Val/Met	− 0.43	− 12.30 to 11.43	0.07
		Val/Val	Reference		
	Adjusted	Met/Met	3.37	− 15.55 to 22.31	0.72
		Val/Met	0.16	− 11.64 to 11.97	0.97
		Val/Val	Reference		
TC	Crud	Met/Met	− 25.42	− 62.49 to 11.64	0.17
		Val/Met	19.56	− 3.64 to 42.77	0.09
		Val/Val	Reference		
	Adjusted	Met/Met	− 22.09	− 59.24 to 15.06	0.24
		Val/Met	21.79	− 1.44 to 45.03	0.06
		Val/Val	Reference		
AIP	Crud	Met/Met	0.07	− 0.05 to 0.20	0.23
		Val/Met	0.12	0.03 to 0.20	**0.004**
		Val/Val	Reference		
	Adjusted	Met/Met	0.06	− 0.06 to 0.20	0.30
		Val/Met	0.12	0.04 to 0.20	**0.003**
		Val/Val	Reference		
AC	Crud	Met/Met	− 0.02	− 0.84 to 0.78	0.94
		Val/Met	0.57	0.05 to 1.08	**0.02**
		Val/Val	Reference		
	Adjusted	Met/Met	− 0.06	− 0.87 to 0.75	0.87
		Val/Met	0.59	0.08 to 1.10	**0.02**
		Val/Val	Reference		
LDL/HDL.CRI.II	Crud	Met/Met	0.23	− 0.12 to 0.59	0.21
		Val/Met	0.05	− 0.16 to 0.28	0.62
		Val/Val	Reference		
	Adjusted	Met/Met	0.27	− 0.08 to 0.62	0.13
		Val/Met	0.05	− 0.16 to 0.27	0.60
		Val/Val	Reference		
TC/HDL.CRI.I	Crud	Met/Met	− 0.25	− 1.10 to 0.59	0.55
		Val/Met	0.20	− 0.72 to 0.31	0.43
		Val/Val	Reference		
	Adjusted	Met/Met	− 0.28	− 1.13 to 0.56	0.51
		Val/Met	− 0.18	− 0.74 to 0.34	0.49
		Val/Val	Reference		

Val/Val genotype is considered as a reference. Low adherence of DTAC is considered as a reference. Generalized linear model; crude model and adjusted model Age, physical activity, sex, smoking, alcohol, energy intake, lipid, and glucose-lowering medicines, and family history of diabetes, as covariates. Bold values denote statistical signifcance at the P < 0.05 level.

*FRAP* ferric reducing ability of plasma, *CI* confidence interval, *AIP* (Atherogenic index of plasma): log(TG/HDL), *AC* (Atherogenic coefficient): (TC-HDL/HDL), *CRI-II* (Castelli s risk index): LDL/HDL, *CRI-I* (Castelli s risk index): TC/HDL, *TC* total cholesterol, *TG* triglyceride, *HDL* high density lipoprotein, *LDL* low density lipoprotein, *WC* waist circumference.

**Table 5:** The interaction of BDNF Val/Met polymorphism and ORAC on anthropometric indices, lipid profile and atherogenic indices.

Variable		Allele	High adherence of ORAC		
			β	95% CI	P
BMI	Crud	Met/Met	1.57	− 0.88 to − 4.02	0.20
		Val/Met	1.69	0.16 to 3.23	**0.03**
		Val/Val	Reference		
	Adjusted	Met/Met	1.62	− 0.82 to 4.06	0.19
		Val/Met	1.84	0.31 to 3.37	**0.01**
		Val/Val	Reference		
WC	Crud	Met/Met	1.75	− 3.91 to 7.43	0.54
		Val/Met	3.60	0.04 to 7.16	**0.04**
		Val/Val	Reference		
	Adjusted	Met/Met	1.50	− 4.17 to 7.19	0.60
		Val/Met	3.75	0.19 to 7.31	**0.03**
		Val/Val	Reference		
HDL	Crud	Met/Met	− 5.50	− 12.20 to 1.19	0.10
		Val/Met	− 4.71	− 8.92 to − 0.51	**0.02**
		Val/Val	Reference		
	Adjusted	Met/Met	− 5.09	− 11.82 to 1.62	0.13
		Val/Met	− 4.62	− 8.83 to − 0.41	**0.03**
		Val/Val	Reference		
TG	Crud	Met/Met	− 0.23	− 57.60 to 57.13	0.99
		Val/Met	44.95	9.09 to 80.82	**0.01**
		Val/Val	Reference		
	Adjusted	Met/Met	− 1.18	− 59.34 to 56.98	0.96
		Val/Met	45.52	9.30 to 81.73	**0.01**
		Val/Val	Reference		
LDL	Crud	Met/Met	− 6.57	− 19.06 to 5.91	0.30
		Val/Met	6.78	− 0.77 to 14.33	0.07
		Val/Val	Reference		
	Adjusted	Met/Met	− 3.46	− 22.29 to 15.37	0.71
		Val/Met	2.44	− 9.33 to 14.22	0.68
		Val/Val	Reference		
TC	Crud	Met/Met	− 27.31	− 63.90 to 9.28	0.14
		Val/Met	13.20	− 9.81 to36.22	0.26
		Val/Val	Reference		
	Adjusted	Met/Met	− 22.16	− 58.97 to 14.63	0.23
		Val/Met	15.15	− 7.92 to 38.22	0.19
		Val/Val	Reference		
AIP	Crud	Met/Met	− 0.03	− 0.16 to 0.09	0.63
		Val/Met	0.09	0.01 to 0.18	**0.01**
		Val/Val	Reference		
	Adjusted	Met/Met	− 0.04	− 0.17 to 0.08	0.50
		Val/Met	0.09	0.01 to 0.17	**0.02**
		Val/Val	Reference		
AC	Crud	Met/Met	− 0.23	− 1.04 to 0.56	0.56
		Val/Met	0.47	− 0.02 to 0.98	0.06
		Val/Val	Reference		
	Adjusted	Met/Met	− 0.26	− 1.07 to 0.53	0.51
		Val/Met	0.43	− 0.07 to 0.93	0.09
		Val/Val	Reference		
LDL/HDL.CRI.II	Crud	Met/Met	0.01	− 0.34 to 0.37	0.94
		Val/Met	0.13	− 0.08 to 0.36	0.23
		Val/Val	Reference		
	Adjusted	Met/Met	0.02	− 0.32 to 0.37	0.88
		Val/Met	0.10	− 0.11 to 0.32	0.36
		Val/Val			
TC/HDL.CRI.I	Crud	Met/Met	0.05	− 0.77 to 0.88	0.90
		Val/Met	− 0.17	− 0.69 to 0.33	0.49
		Val/Val	Reference		
	Adjusted	Met/Met	0.01	− 0.82 to 0.85	0.96
		Val/Met	− 0.16	− 0.68 to 0.35	0.53
		Val/Val	Reference		

Val/Val genotype is considered as a reference. Low adherence of DTAC is considered as a reference. Generalized linear model; crude model and adjusted model Age, physical activity, sex, smoking, alcohol, energy intake, lipid, and glucose-lowering medicines, and family history of diabetes, as covariates. Bold values denote statistical signifcance at the P < 0.05 level.

*ORAC* oxygen radical absorbance capacity, *CI* confidence interval, *AIP* (Atherogenic index of plasma): log(TG/HDL), *AC* (Atherogenic coefficient): (TC-HDL/HDL), *CRI-II* (Castelli s risk index): LDL/HDL, *CRI-I* (Castelli s risk index): TC/HDL, *TC* total cholesterol, *TG* triglyceride, *HDL* high density lipoprotein, *LDL* low density lipoprotein, *WC* waist circumference.

**Table 6:** The interaction of BDNF Val/Met polymorphism and TEAC on anthropometric indices, lipid profile and atherogenic indices.

Variable		Allele	High adherence of TEAC		P
			β	95% CI	
BMI	Crud	Met/Met	− 7.09	− 13.77 to − 0.24	0.04
		Val/Met	− 1.82	− 6.02 to 2.38	0.39
		Val/Val	Reference		
	Adjusted	Met/Met	1.60	− 0.85 to 4.05	0.20
		Val/Met	1.31	− 0.19 to 2.83	0.08
		Val/Val	Reference		
WC	Crud	Met/Met	− 20.43	− 57.26 to 16.40	0.27
		Val/Met	22.44	− 0.52 to 45.42	0.05
		Val/Val	Reference		
	Adjusted	Met/Met	3.15	− 2.40 to 8.70	0.26
		Val/Met	2.31	− 1.13 to 5.76	0.18
		Val/Val	Reference		
HDL	Crud	Met/Met	− 7.09	− 13.77 to − 0.24	**0.04**
		Val/Met	− 1.82	− 6.02 to 2.38	0.39
		Val/Val	Reference		
	Adjusted	Met/Met	− 6.03	− 12.64 to 0.56	0.07
		Val/Met	− 5.67	− 5.79 to 2.41	0.41
		Val/Val	Reference		
LDL	Crud	Met/Met	− 5.99	− 24.91 to 12.93	0.53
		Val/Met	− 1.65	− 13.43 to 10.11	0.78
		Val/Val	Reference		
	Adjusted	Met/Met	− 1.42	− 20.29 to 17.44	0.88
		Val/Met	− 1.30	− 13.02 to 10.41	0.82
		Val/Val	Reference		
TC	Crud	Met/Met	− 20.43	− 57.26 to 16.40	0.27
		Val/Met	22.44	− 0.52 to 45.42	0.05
		Val/Val	Reference		
	Adjusted	Met/Met	− 17.15	− 54.06 to 19.75	0.36
		Val/Met	26.39	3.40 to 49.38	**0.02**
		Val/Val	Reference		
TG	Crud	Met/Met	− 30.82	− 88.36 to 26.71	0.29
		Val/Met	53.51	17.80 to 89.22	**0.003**
		Val/Val	Reference		
	Adjusted	Met/Met	− 32.04	− 90.16 to 26.08	0.28
		Val/Met	55.85	19.85 to 91.84	**0.002**
		Val/Val	Reference		
AIP	Crud	Met/Met	0.005	− 0.12 to 0.13	0.94
		Val/Met	0.105	0.02 to 0.18	**0.01**
		Val/Val	Reference		
	Adjusted	Met/Met	− 0.002	− 0.13 to 0.12	0.98
		Val/Met	0.10	0.02 to 0.18	**0.009**
		Val/Val	Reference		
AC	Crud	Met/Met	0.03	− 0.77 to 0.83	0.93
		Val/Met	0.51	0.01 to 1.02	**0.04**
		Val/Val	Reference		
	Adjusted	Met/Met	0.02	− 0.78 to 0.82	0.96
		Val/Met	0.55	0.05 to 1.06	**0.03**
		Val/Val	Reference		
LDL/HDL.CRI.II	Crud	Met/Met	0.10	− 0.25 to 0.47	0.55
		Val/Met	− 0.04	− 0.26 to 0.17	0.69
		Val/Val	Reference		
	Adjusted	Met/Met	0.15	− 0.20 to 0.50	0.39
		Val/Met	− 0.05	− 0.27 to 0.16	0.64
		Val/Val	Reference		
TC/HDL.CRI.I	Crud	Met/Met	0.25	− 0.58 to 1.09	0.55
		Val/Met	− 0.04	− 0.56 to 0.46	0.86
		Val/Val	Reference		
	Adjusted	Met/Met	0.22	− 0.61 to 1.06	0.60
		Val/Met	− 0.03	− 0.54 to 0.48	0.91
		Val/Val	Reference		

Val/Val genotype is considered as a reference. Low adherence of DTAC is considered as a reference. Generalized linear model; crude model and adjusted model Age, physical activity, sex, smoking, alcohol, energy intake, lipid, and glucose-lowering medicines, and family history of diabetes, as covariates. Bold values denote statistical signifcance at the P < 0.05 level.

*TEAC* trolox equivalent antioxidant capacity, *CI* confidence interval, *AIP* (Atherogenic index of plasma): log(TG/HDL), *AC* (Atherogenic coefficient): (TC-HDL/HDL), *CRI-II* (Castelli s risk index): LDL/HDL, *CRI-I* (Castelli s risk index): TC/HDL, *TC* total cholesterol, *TG* triglyceride, *HDL* high density lipoprotein, *LDL* low density lipoprotein, *WC* waist circumference.

**Table 7:** The interaction of BDNF Val/Met polymorphism and TRAP on anthropometric indices, lipid profile and atherogenic indices.

Variable		Allele	High adherence of TRAP		
			β	95% CI	P
BMI	Crud	Met/Met	0.87	− 1.61 to 3.36	0.49
		Val/Met	1.20	− 0.32 to 2.73	0.12
		Val/Val	Reference		
	Adjusted	Met/Met	0.81	− 1.66 to 3.29	0.52
		Val/Met	1.81	− 0.33 to 2.69	0.12
		Val/Val	Reference		
WC	Crud	Met/Met	0.88	− 4.86 to 6.63	0.76
		Val/Met	3.75	0.20 to 7.29	**0.03**
		Val/Val	Reference		
	Adjusted	Met/Met	0.93	− 4.66 to 6.53	0.74
		Val/Met	3.36	− 0.07 to 6.80	0.05
		Val/Val	Reference		
HDL	Crud	Met/Met	− 3.58	− 10.40 to 3.23	0.30
		Val/Met	− 3.86	− 8.06 to 0.34	0.07
		Val/Val	Reference		
	Adjusted	Met/Met	− 3.16	− 9.83 to 3.49	0.35
		Val/Met	− 3.71	− 7.80 to 0.38	0.07
		Val/Val	Reference		
TC	Crud	Met/Met	− 18.56	− 55.53 to 18.40	0.32
		Val/Met	34.93	12.10 to 57.76	**0.003**
		Val/Val	Reference		
	Adjusted	Met/Met	− 18.29	− 55.35 to 18.76	0.33
		Val/Met	37.20	14.38 to 60.03	**0.001**
		Val/Val	Reference		
TG	Crud	Met/Met	− 33.52	− 91.54 to 24.48	0.25
		Val/Met	63.78	28.09 to 99.46	** < 0.001**
		Val/Val	Reference		
	Adjusted	Met/Met	− 37.97	− 96.61 to 20.67	
		Val/Met	65.28	29.36 to 101.20	** < 0.001**
		Val/Val	Reference		
LDL	Crud	Met/Met	1.37	− 17.70 to 20.46	0.88
		Val/Met	1.39	− 10.36 to 13.14	0.81
		Val/Val	Reference		
	Adjusted	Met/Met	5.53	− 13.50 to 24.56	0.56
		Val/Met	1.21	− 10.46 to 12.90	0.83
		Val/Val	Reference		
AIP	Crud	Met/Met	− 0.01	− 0.14 to 0.11	0.85
		Val/Met	0.16	0.07 to 0.24	** < 0.001**
		Val/Val	Reference		
	Adjusted	Met/Met	− 0.02	− 0.15 to 0.11	0.76
		Val/Met	0.16	0.08 to 0.24	** < 0.001**
		Val/Val	Reference		
AC	Crud	Met/Met	− 0.12	− 0.93 to 0.68	0.76
		Val/Met	0.88	0.38 to 1.38	**0.001**
		Val/Val	Reference		
	Adjusted	Met/Met	− 0.15	− 0.96 to 0.65	0.71
		Val/Met	0.89	0.39 to 1.39	** < 0.001**
		Val/Val	Reference		
LDL/HDL.CRI.II	Crud	Met/Met	0.14	− 0.21 to 0.51	0.42
		Val/Met	0.09	− 0.13 to 0.31	0.41
		Val/Val	Reference		
	Adjusted	Met/Met	0.19	− 0.15 to 0.55	0.27
		Val/Met	0.07	− 0.14 to 0.29	0.48
		Val/Val	Reference		
TC/HDL.CRI.I	Crud	Met/Met	− 0.25	− 1.10 to 0.60	0.56
		Val/Met	− 0.21	− 0.72 to 0.30	0.41
		Val/Val	Reference		
	Adjusted	Met/Met	− 0.23	− 1.09 to 0.61	0.58
		Val/Met	− 0.21	− 0.73 to 0.30	0.41
		Val/Val	Reference		

Val/Val genotype is considered as a reference. Low adherence of DTAC is considered as a reference. Generalized linear model; crude model and adjusted model Age, physical activity, sex, smoking, alcohol, energy intake, lipid, and glucose-lowering medicines, and family history of diabetes, as covariates. Bold values denote statistical signifcance at the P < 0.05 level.

*TRAP* total reactive antioxidant potential, *CI* confidence interval, *AIP* (Atherogenic index of plasma): log(TG/HDL), *AC* (Atherogenic coefficient): (TC-HDL/HDL), *CRI-II* (Castelli s risk index): LDL/HDL, *CRI-I* (Castelli s risk index): TC/HDL, *TC* total cholesterol, *TG* triglyceride, *HDL* high density lipoprotein, *LDL* low density lipoprotein, *WC* waist circumference.

**Figure 1:**
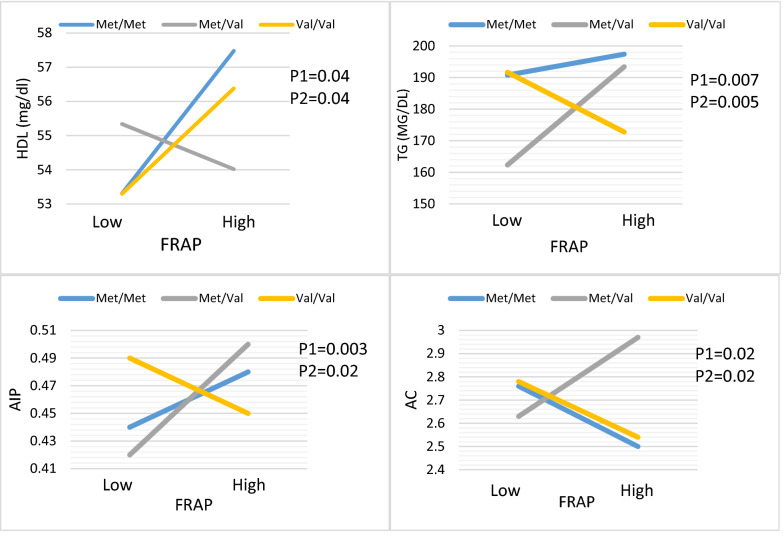
Interaction between the BDNF Val66Met polymorphism and DTAC (FRAP) intake with regard to TG, HDL, AIP and AC according to the median DTAC, the participants were dichotomized into low and high categories. P 1 = P value with unadjusted (crude) model, P 2 = P value with adjustments for potential confounding factors including (Age, physical activity, sex, smoking, alcohol, energy intake, lipid, and glucose-lowering medicines, and family history of diabetes).

In both crud and adjusted model for potential confounders, BDNF Val66Met and **FRAP** interactions were significant in terms of HDL, TG, AIP and AC index. Carriers of the Val/Met genotype who were in the higher median intake of FRAP had lower HDL (β: − 4.18, 95%CI − 8.29 to − 0.68, P:0.04) and higher TG concentration (β: 51.84, 95%CI 15.39 to 88.30, P:0.005), AIP (β: 0.12, 95%CI 0.04 to 0.20, P:0.003), and AC (β: 0.59, 95%CI 0.08 to 1.10, P:0.02) index compared to Val/Val genotypes whit lower median intake (Table 4, Fig. 1).

**Figure 2:**
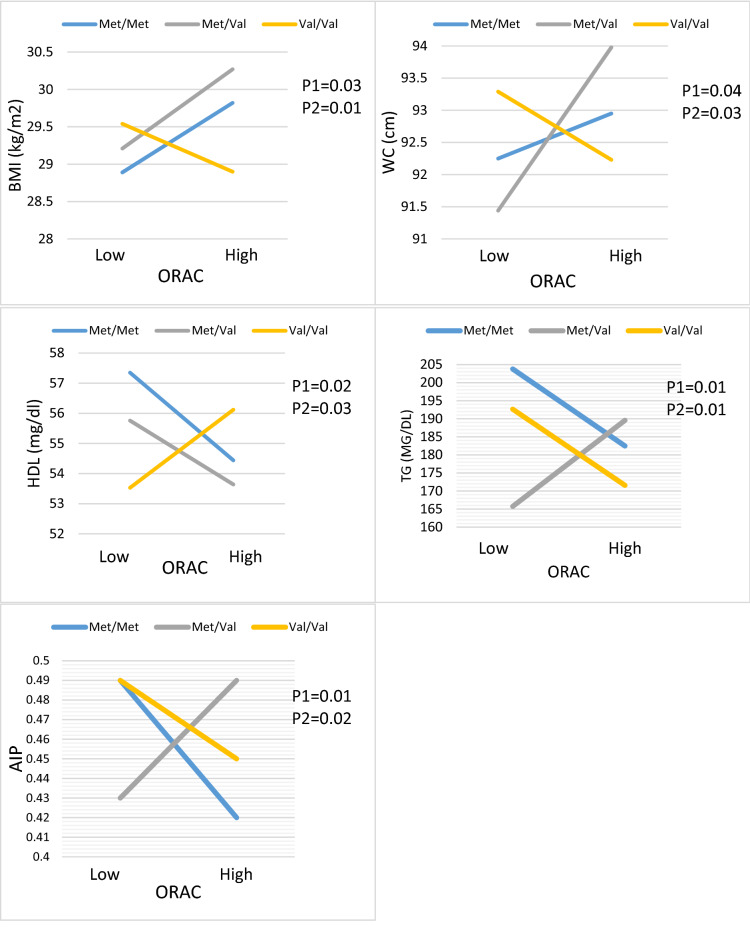
Interaction between the BDNF Val66Met polymorphism and DTAC (ORAC) intake with regard to TG, HDL, AIP and AC according to the median DTAC, the participants were dichotomized into low and high categories. P 1 = P value with unadjusted (crude) model, P 2 = P value with adjustments for potential confounding factors including (Age, physical activity, sex, smoking, alcohol, energy intake, lipid, and glucose-lowering medicines, and family history of diabetes).

Besides, BDNF Val66Met and **ORAC** interactions were significant in terms of anthropometric indices (BMI and WC), lipid profiles (HDL and TG), and atherogenic index (AIP) in crude model and after adjusted to cofounding factors. Diabetic patients with Val/Met genotype who consumed higher ORAC intake had increased odds for BMI (β: 1.84, 95%CI 0.31 to 3.37, P:0.01), WC (β: 3.75, 95%CI 0.19 to 7.31, P:0.03), TG (β: 45.52, 95%CI 9.30 to 81.73, P:0.01) and AIP (β: 0.09, 95%CI 0.01 to 0.17, P:0.02), also decreased odds for HDL concentration (β: − 4.62, 95%CI − 8.83 to − 0.41, P:0.03) compared to reference group whit lower ORAC intake (Table 5, Fig. 2).

**Figure 3:**
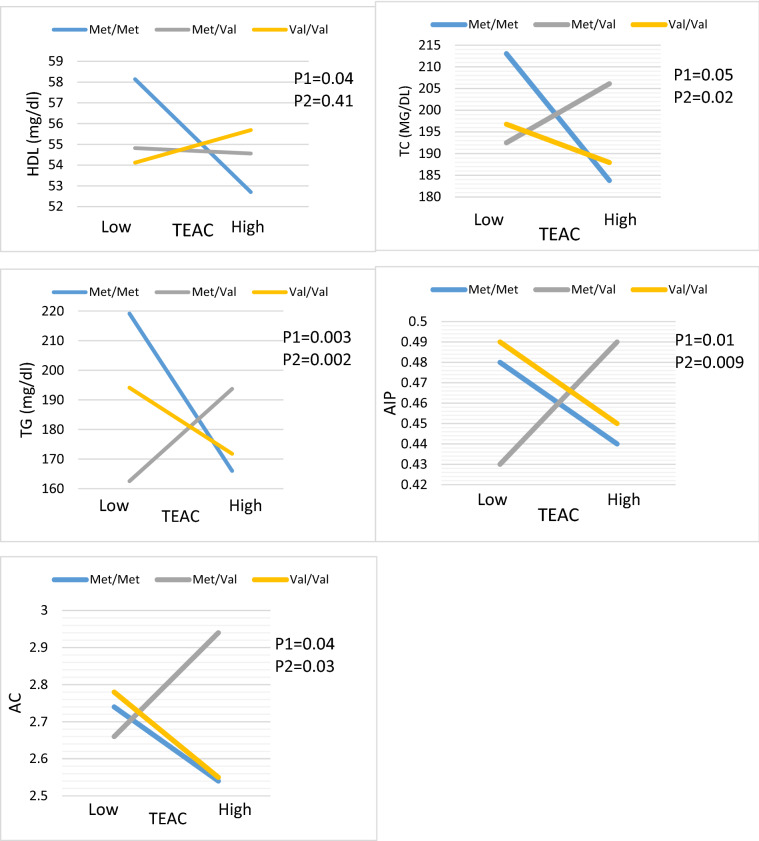
Interaction between the BDNF Val66Met polymorphism and DTAC (TEAC) intake with regard to TG, HDL, AIP and AC according to the median DTAC, the participants were dichotomized into low and high categories. P 1 = P value with unadjusted (crude) model, P 2 = P value with adjustments for potential confounding factors including (Age, physical activity, sex, smoking, alcohol, energy intake, lipid, and glucose-lowering medicines, and family history of diabetes).

Moreover, Val/Met patients with a higher median intake of **TEAC** had higher TG (β: 55.85, 95%CI 19.85 to 91.84, P = 0.002), AIP (β: 0.10, 95%CI 0.02 to 0.18, P = 0.009) and AC (β: 0.55, 95%CI 0.05 to 1.06, P = 0.03) compared to reference group whit lower TEAC intake in both crud and adjusted model. Moreover, in the crude model, there was no significant interaction between Val/Met group and TEAC intake on TC (β: 22.44, 95% CI − 0.52 to 45.42, P:0.05) however, after controlling for confounders, a significant interaction was found on TC (β: 26.39, 95% CI 3.40 to 49.38, P:0.02). In crude model, Met/Met group with higher TEAC intake had lower HDL (β: − 7.09, 95% CI − 13.77 to − 0.24, P:0.04), also after adjustment for cofounders this association was disappeared (β: − 6.03, 95% CI − 12.64 to 0.56, P:0.07) (Table 6, Fig. 3).

**Figure 4:**
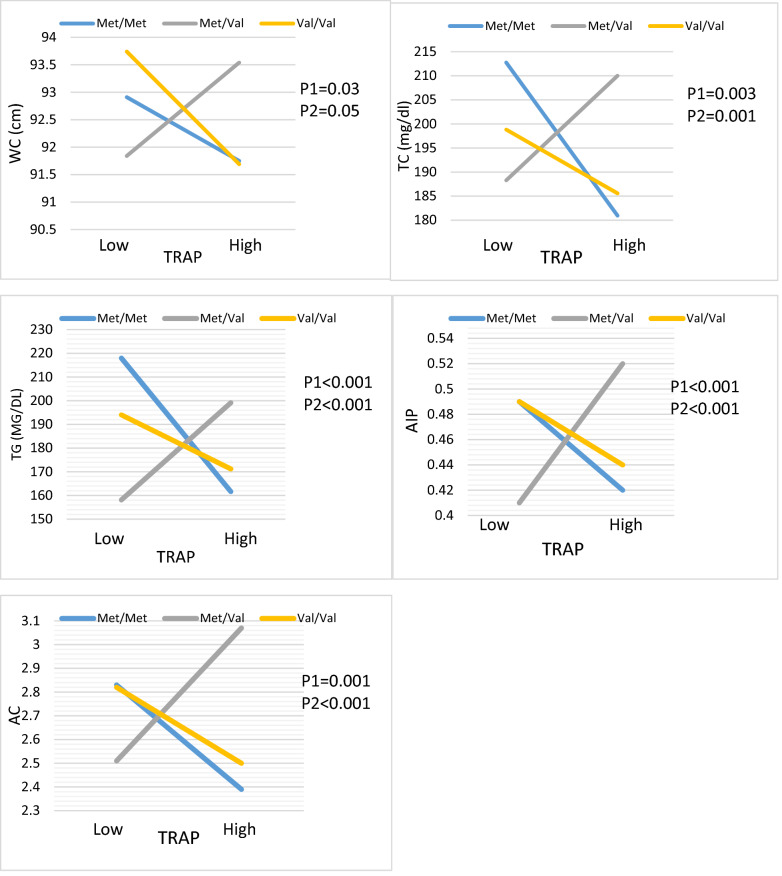
Interaction between the BDNF Val66Met polymorphism and DTAC (TRAP) intake with regard to TG, HDL, AIP and AC according to the median DTAC, the participants were dichotomized into low and high categories. P 1 = P value with unadjusted (crude) model, P 2 = P value with adjustments for potential confounding factors including (Age, physical activity, sex, smoking, alcohol, energy intake, lipid, and glucose-lowering medicines, and family history of diabetes).

Finally, BDNF Val66Met and **TRAP** interactions were significant in terms of the anthropometric index (WC), lipid profiles (TC and TG), and atherogenic indices (AIP and AC). Diabetic patients with Val/Met genotype who consumed higher TRAP intake had increased odds for TC (β: 37.20, 95%CI 14.38 to 60.03, P:0.001), TG (β: 65.28, 95%CI 29.36 to 101.20, P < 0.001), AIP (β: 0.16, 95%CI 0.08 to 0.24, P < 0.001) and AC (β: 0.89, 95%CI 0.39 to 1.39, P < 0.001) compared to reference group whit lower TRAP intake in both crud and adjusted model (Table). Moreover, in the crude model, there was a significant interaction between the Val/Met group in comparison with the reference group (Val/Val) on WC (β: 3.75, 95%CI 0.20 to 7.29, P:0.03), however, after controlling for confounders, a significant interaction was disappeared WC (β: 3.36, 95%CI − 0.07 to 6.80, P:0.05) (Table 7, Fig. 4).

## Discussion

The key findings of the current study were the significant interaction result of BDNF Val66Met polymorphism with DTAC on lipid profile and atherogenic indices in T2DM patients. High DTAC intake modified the association of the BDNF Val66Met genotypes with the odds of higher lipid profile and atherogenic indices. Particularly, we revealed that increased DTAC did not influence the negative consequences of the Val/Met genotype. While it seems that the presence of the Val/Val wildtype and BDNF Met/Met homozygotes in diabetic patients with a high DTAC is a protective factor.

In this present study, we revealed that an individual with higher adherence to DTAC had lower TAC concentrations. Moreover, patients with a higher intake of TRAP were more likely to have higher IL-18 concentrations. There have been few investigations on the relationship between DTAC and metabolic indicators such as lipid profiles and inflammatory markers; the findings of numerous research have contradicted the findings of the current study^20,31–35^. In line with our findings, Mozaffari et al.^36^, have shown subjects in the highest tertile of dietary TRAP had higher BMI than those in the lowest tertile. Numerous findings have reported obesity has been linked to a persistent low-grade inflammatory disease, which has been linked to the development of T2DM and CVD^37,38^. In these situations, human adipose tissue secretes a high amount of inflammatory markers, including IL-18, in these circumstances^39^.

We did not find significant association between BDNF rs6267 groups and biochemical markers. BDNF play important role in etiology of diabetes and obesity by probably biological mechanism including controlling food behaviour, energy homeostasis and anorexigenic effects^40^. Some experimental studies have also suggested that hyperphagia, hyperinsulinemia, and higher levels of serum leptin and body weight following a decrease in BDNF levels, among BDNF-knockout mice^41^. In terms of human study, association between Val66Met polymorphism with obesity, dyslipidaemia and diabetes are controversial. For instance, several studies have shown that Met/Met genotype have higher risk for insulin resistance, obesity and dyslipidaemia^24,42,43^. Bonaccorso et al. revealed, Met-allele of BDNF Val66Met polymorphism was to be positively associated with serum levels TG and TG/HDL-C ratio^24^. However, some authors have suggested the Met-allele carriers have lower risk for obesity, postprandial glucose and HbA1c levels^25,44,45^. Additionally, also some studies have observed no association with anthropometric indices and lipid profile^46–48^. The inconsistent results have been revealed on the association between the Val66Met polymorphism and obesity and lipid profile, proposing that environmental factors like dietary intake may be modify this association.

In this study, we discovered that a diet high in overall antioxidant capacity is more beneficial in homozygotes carriers than in Val/Met carriers. This is the first research to investigate at how dietary antioxidants interact with the BDNF polymorphism. In addition, only a few studies have examined the relationship between BDNF rs6267 and dietary patterns, as well as food and nutrient intakes^29,30^. According to a pervious study, individuals with BDNF Val/Met and Met/Met had a lower risk for T2DM in low energy intake and especially BDNF Val/Met had a negative association with low-protein, high-carbohydrate, and low-fat diet. In comparison to BDNF Val/Val, BDNF Val/Met reduced the risk of HOMA-IR in low-energy intake but raised the risk of HOMA-B in high-energy intake. HOMA-B is an insulin secretion capacity index that could aim to decrease the incidence of T2DM. When high-energy consumption is combined with BDNF Val/Met, insulin secretion is increased in terms of maintaining normoglycemia condition. As a result, individuals with BDNF Val/Met may also have a higher potential to compensate for the development of T2DM^29^. In this term, another study has shown, total food intake, total caloric intake, and protein intake were not related to the BDNF rs6265 variation. Regarding obesity indicators, although, this variation interacted with PUFA and total food intake. Met allele carriers in men exhibited a higher BMI as their PUFA intake increased, and a smaller waist as their n-3: n-6 PUFA ratio increased. In contrast to heterozygotes, Val/Val homozygous men showed the opposite trend in BMI: BMI dropped with increased PUFA intake and higher n-3:n-6 PUFA ratio increased waist circumference^30^. Furthermore, another study indicated that when Met allele carriers were exposed to a high-CHO diet, their chance of developing carbohydrate-induced hypertriglyceridemia enhanced^49^. In contrast, another study has shown, Val66Met polymorphism did not appear to affect the link between food quality and BDNF serum in terms of depression prediction^50^. Previous research has revealed that dietary intakes alter the relationship between BDNF genotype and obesity-related behaviors, which is corroborated by findings in rats^51–53^. As a result, food consumption may influence the relationship between BDNF polymorphism and cardiovascular disease indicators via BDNF expression and serum protein modulation.

On the effect of diet on the BDNF serum, some nutrigenomic studies have done based on diet or other macronutrient induced obesity and healthy diet. For example, a previous study found that high glucose concentrations decreased BDNF release^54^. As a result, blood BDNF levels in T2DM patients were shown to be considerably higher than in healthy controls in humans and were found to be strongly associated with triglyceride levels^55^. In diabetic mice, subcutaneous injections of BDNF were found to considerably improve lipid and glucose profiles^56^. In this regard, experimental studies have revealed that diet-induced obese mice include high-fat and high-sugar diets^53,57,58^ or n-3 PUFA deficient diets^51^ and chronic high-fat DIO mice^53^ lowered BDNF expression in the hippocampus by more than 30%, which has been associated to weaker inhibitory regulation of food consumption and, as a result, promoted obesity-related phenotype, while low-fat mice showed no difference.

We observed that the presence of the Val/Val wild-type and BDNF Met/Met homozygotes in diabetic patients with a high DTAC is a protective factor. Antioxidant-rich diets may affect previously unknown biological processes, altering vulnerability to cardiovascular disease. Antioxidant-rich diets have been demonstrated to have a positive influence on metabolic syndrome components, cardiovascular disease risk factors, and obesity-related aspects in several epidemiological investigations^36,59,60^. Reduced inflammation and oxidative stress, increased leptin gene expression, appetite regulation, adipocyte metabolism regulation, and suppression of nuclear factor-B factor are all probable mechanisms^36,61,62^.

We investigated the nutrigenomic research on BDNF serum because there was no nutrigenetics study in this term. In adult rats, fish oil treatment resulted in a considerable increase in BDNF expression^52^. Furthermore, administration of whole-grain (WG) rye has been demonstrated to upregulate BDNF levels. When compared to white wheat flour-based bread meals raised BDNF levels by 27% after fasting^63^. Furthermore, prebiotic feeding elevated the expression of BDNF and peptide YY^64^. Furthermore, there was a ‘very probable' rise in BDNF levels with protein supplementation^65^. A Mediterranean-style diet^66^, omega-3 fatty acids^67,68^, and even vitamin E and refined flavonoids consumption have all been associated with increased concentrations of brain BDNF. There are some possible mechanisms for these favourable effects in our study and nutrigenomic studies include similar components. Endothelial dysfunction is prevented by a high antioxidant diet, which is linked to reduced levels of pro-inflammatory cytokines in the plasma^69–72^ Moreover, pro-inflammatory cytokines like IL-6 and TNF- may also suppress BDNF expression^73^. Compliance to these interventions would be predicted to be related with greater plasma BDNF concentrations under this mechanism. These antioxidant components increase BDNF levels and phosphorylation of the CREB pathway^74–76^. Although we observed being in BDNF Val/Val wild-type and BDNF Met/Met homozygotes reduced lipid markers and atherogenic indices between homozygotes participant, heterozygotes have shown increased these factors even in high DTAC. These results go beyond previous reports, showing the negative effect of heterozygotes BDNF mic opposite of homozygotes. The heterozygotes BDNF val66met genotype is correlated with cortical morphology that differs from that of BDNF val66met homozygotes. The BDNF Val/Met genotype, in particular, may affect brain tissue volumes and neurodevelopment, resulting in phenotypic differences between BDNF Val/Val wildtype and BDNF Met/Met homozygotes^77–79^. Interestingly, 5-HT turnover was impaired in heterozygous BDNF+/− mice with lowered BDNF expression and resulting in increased food intake and obese phenotypes^80^. Kernie et al. found that heterozygous BDNF/– mice with low BDNF mRNA expression gain 300% more body fat and develop obesity than homozygous BDNF/– mice with high BDNF mRNA expression^81^. Consequently, diet-induced dyslipidemia may be exacerbated by the downregulation of BDNF in heterozygotes individuals. Besides, these discrepancies may be attributed to variations in food choices, fortification, and preferences, which could also impact antioxidant intake through various dietary sources, as well as some changes in antioxidant activity and availability that occur during food processing and preparation^82–85^. The unfavorable impact found in our study between Val/Met genotypes could be related to a higher intake of high-calorie antioxidant-rich foods and a higher energy intake, since we discovered in our study that greater DTAC consumption was associated with higher carbohydrate intake in diabetic patients^86^. We did not separate different sources of TAC, also based on previous studies source of TAC may have significant effect on biochemical markers^87^.

## Limitations and strengths

limitations of the present study including the cross-sectional design, so any causality cannot be argued; the use of FFQ for dietary assessing. Due to financial limitations, it was not possible to perform a western blot analysis to determine whether rs6265 SNP alters the expression of BDNF. markers. Furthermore, our participants were from the Iranian country which may not be generalized due to racial and regional differences. Despite the limitations mentioned above, this is the first effort to study the interaction between BDNF Val66Met polymorphism and DTAC on lipid profiles and atherogenic indices. Recognition of these gene-diet interactions could be determining in prescribe personalized nutritional recommendations for the improvement and management of CVD risk in T2DM patients. Finally, these results can be used in combination with a patient’s genetic history to provide more applicable and tailored nutritional advice for preventing or attenuating cardiovascular disease in T2DM patients.

## Conclusion

However, this study has several strengths among which it should be emphasized that is the first study of the gene-environment interaction in diabetic patients exploring how BDNF polymorphism (Val66Met) affects the diet in correlation with lipid profiles and atherogenic indices, adding important information to previous studies that assessed dietary habits and lipid markers in diabetic patient’s groups without considering genetic implications. Further functional studies are necessary to confirm the exact mechanism through which this SNP influences food intake regulation.

## Method

### Study population

The current cross-sectional study was carried out on 667 T2DM patients who were referred from diabetes referral clinics in Tehran, Iran. Our study comprised diabetic patients with fasting blood sugar levels of > 126 mg/dl or who were on glucose-lowering medicines. The complete inclusion and exclusion criteria, demographic, physical activity (METs) information, and anthropometric measurements (body mass index (BMI) and WC), were taken based on our previous larger investigation^88^. The International Physical Activity Questionnaire (IPAQ) short form was used to assess physical activity. The reliability and validity of the IPAQ has previously been evaluated in Iranian adolescents^89^. All of the patients were asked to give their informed permission. The study was conducted based on the Declaration of Helsinki, and Ethics Committee of the Tehran University of Medical Sciences approved the protocol (no. 15060).

### Biochemical assessments

The study participants' venous blood samples were taken after they had fasted for 12 h. The levels of HDL-C and LDL-C in the blood were measured using a Roche Hitachi analyzer using turbidimetry (Roche, Germany). The ELISA approach was also used to determine the serum levels of leptin and ghrelin (Bioassay Technology Co, China and Mediagnost, Germany, respectively). The number of inflammatory markers in the blood, such as IL-18 and PTX3, was measured using the ELISA method (Shanghai Crystal Day Biotech Co., Ltd). The intra-assay and interassay coefficients of variation (CV) were less than 10% and 12%, respectively, for the IL-18 ELISA kit, which had a sensitivity of 28 ng/l. The intra-assay and interassay CVs were less than 10% and 12%, respectively, for the PTX3 ELISA kit, which has a sensitivity of 0.05 ng/ml. The levels of hs-CRP in the blood were measured using an ELISA kit (Diagnostic Biochem Canada Inc., London, Ontario, Canada). The intra-assay and interassay CVs were both less than 5% and 9.5%, respectively. Both the intra- and inter-assay CVs were less than 5% and 9.5%, respectively. The total antioxidant capacity of the serum was determined using specttrophometry (TAC). The serum enzymatic activity of SOD was measured using a colorimetric method (Cayman Chemical Company, USA). The concentration of 8-isoprostane F2 in the blood was measured using an ELISA (Shanghai Crystal Day Biot). The Nutrition and Genomics Laboratory at TUMS was used to conduct all of the tests.

### Atherogenic indices of plasma (AIP) and lipid ratio assessment

The atherogenic indices of plasma were calculated using the logarithmic ratio of (TG to HDL-C) (AIP). Furthermore, Olamoyegun et al. invented the lipid ratio, which is calculated using the following formula: **CRI-I** = TC/HDL-C, **CRI-II** = LDL-C/HDL-C, **AC** = (TC − HDL-C)/HDL-C.

### Dietary assessment and DTAC calculation

Dietary data were analyzed using a standard semi-quantitative FFQ that included 147 food categories and was specifically prepared for usage in Iran^90^. Face-to-face personal interviews with professional dietitians were used to complete the FFQ. Participants were asked to rate the frequency with which they consumed each food item throughout the previous year. Using household measures, the portion sizes of ingested food products were translated to grams per day. The Total Antioxidant Capacity (TAC) is an indicator of overall plasma antioxidant status consisting of four indices: TEAC and TRAP relying on Italian food databases^91^, FRAP based on Norwegian antioxidant table and reduce ferric iron to ferrous iron^92^, and ORAC based on United States Department of Agriculture (USDA) databases, that also demonstrates the sample's capacity to exchange hydrogen to stabilize a free radical^93^.

### DNA extraction and gene sequencing

For DNA extraction, a salting-out approach was applied^20^. The PCR–RFLP method was used to genotype the Val66Met polymorphism. The following primers were used to amplify rs6265: Forward: 5′-CACTAGCCCAGAGAGAGGAGTG-3′, Revers:50-TGAGCCCAGCCGCACACTAAC. 75 ng genomic DNA, 0.6 mM of each primer, and 2X Taq DNA Polymerase Master Mix were included in the final volume of the PCR result (Amplicon; Germany). Denaturation at 95 °C for the 30 s (40 cycles), annealing at 8 °C for 30 s, and 40 s of extension at 72 °C were used in the PCR cycles, with a final extension at 65 °C for 30 min. Finally, the products were electrophoresed on 2% agarose gels. Importantly, 15% of the samples were directly sequenced for confirmation of the PCR–RFLP results. The sequencing process performed using the ABI PRISM 3730 automated sequencer (Applied Biosystems, Foster City, Calif, USA).

### Statistical analyses

The Kolmogorov–Smirnov test was used to determine the data's normality. In this study, the sample size was calculated according to following formula: N = (([(Z1 − α + Z1 − β) × √1 − r 2]/r) 2 + 2), whit considering r = 0.15, β = 0.95 and α = 0.05. The Hardy–Weinberg equilibrium (HWE) was assessed with the χ^2^ test. Based on their FRAP, TRAP, TEAC, and ORAC scores, the subjects were separated into two groups: low and high intakes. Qualitative variables were compared with one-way ANOVA and analysis of covariance (ANCOVA) in crude and adjusted models respectively. Potential interactions between the rs6265 genotype and DTAC on lipid profiles and atherogenic indices were investigated using the generalized linear models (GLMs) model. Age, physical activity, sex, smoking, alcohol, energy intake, lipid, and glucose-lowering medicines, and family history of diabetes were all used as cofounder factors in adjusted analyses. All stages of our research's analysis were conducted using SPSS software (SPSS Inc., Chicago, IL, USA, version 25). A p-value of less than 0.05 was also considered significant.

### Ethics approval and consent to participate

The protocol of the study was approved by the ethics committee of TUMS. All participants completed a written informed consent.
